# Study on preparation and sintering properties of nano-silver-coated tin slurry

**DOI:** 10.1098/rsos.221492

**Published:** 2023-06-07

**Authors:** Hui Yang, Huan You

**Affiliations:** ^1^ Education Department of Guangxi Zhuang Autonomous Region, Key Laboratory of Advanced Manufacturing and Automation Technology (Guilin University of Technology), Guilin 541006, People's Republic of China; ^2^ College of Mechanical and Control Engineering, Guilin University of Technology, Guilin 541006, People's Republic of China

**Keywords:** nano-silver-coated tin, heterogeneous flocculation method, strength of sintering

## Abstract

A nano-sized silver-coated tin (Sn@Ag) slurry was prepared by heterogeneous flocculation method by adjusting the pH value of solution and selecting different dispersants. The slurry improved the oxidation resistance of tin and its dispersibility in silver matrix. The sintering strength of nanometre Sn@Ag slurry increases with the increase of Sn content. When the Sn content reaches 5%, the shear strength of the joint reaches the highest 50 MPa, which is more than 10 MPa higher than that of the pure nanometre silver slurry sintered joint. The increase of shear strength is due to the fact that the equilibrium phase formed after sintering is Ag–Sn replacement solid solution and intermetallic compound Ag_3_Sn, which have the effect of replacement solution strengthening and dispersion strengthening, respectively. It is proved by experiments and analysis that the application of nano-silver paste in chip interconnection is feasible. The research of this subject provides experimental reference and theoretical basis for the application of new generation interconnect materials in power devices and promotes the development of microelectronics packaging technology.

## 1. Introduction

The rapid growth of silicon carbide semiconductor technology requires new high-performance materials to build low-loss, high-density and heat-stable integrated packages. As an electronic packaging interconnect material, nano-silver paste can be used at high temperature instead of traditional solder, which is suitable for multi-stage packaging systems [1–3].

In order to apply nano-silver paste to power device chip interconnections, the main problems to be solved are as follows: (i) how to ensure the uniform and controllable coating of nano-silver paste to the high-density micrometric convex points; (ii) the sintering temperature of nano-silver slurry is high and the sintering strength is low [4], how to effectively improve its sintering performance; and (iii) how to improve the reliability of nano-silver solder joints when flip chips are in high-temperature state for a long time.

Reducing the diameter of nano-Ag particles can achieve sintering at a lower temperature, but this will lead to variable production process and increased cost [5], which is not suitable for industrial mass production. Based on the theory of transient liquid phase sintering, the sintering temperature can be effectively reduced by doping low melting point tin into nano-silver. The intermetallic compound Ag_3_Sn generated after sintering is a hard and brittle phase, which plays a role of dispersion strengthening, so as to improve the shear strength and reliability of solder joints. However, the doped tin is easily oxidized in the air, so the scheme of nano-silver-coated tin core–shell structure (Sn@Ag) is adopted.

Heterogeneous flocculation has the advantages of mild reaction conditions and less equipment investment. It is more used in improving the performance of ceramic materials but rarely used in electronic packaging interconnect materials. Therefore, it is a new attempt to prepare Sn@Ag core–shell nanoparticles by heterogeneous flocculation. Heterogeneous flocculation method was used to prepare Sn@Ag core–shell nanoparticles by selecting different types of surfactants and adjusting the pH value of the solution, so as to improve the oxidation resistance of tin and dispersibility in silver matrix. According to the thermal decomposition temperature of dispersant and organic solvent, sintering process was developed and sintering experiment was conducted. The change of sintering strength of nanometre Sn@Ag slurry with the increase of Sn content was studied, and the strengthening mechanism of core–shell structure and sintering properties of samples were analysed by scanning electron microscopy (SEM) and X-ray diffraction (XRD) techniques.

## 2. Preparation of nano-silver-coated tin slurry

In order to prepare nano-Sn@Ag core–shell materials by heterogeneous flocculation method, first, silver nanoparticles and tin nanoparticles were adsorbed by negative and cationic dispersants, respectively. Silver nanoparticles have higher isoelectric point, anionic dispersants can have stronger adsorption on silver nanoparticles and tin nanoparticles have lower isoelectric point, and cationic dispersants can be adsorbed on tin nanoparticles in large quantities. Second, according to experience, the particle size of the ‘nuclear layer’ powder is generally much larger than that of the ‘shell layer’ powder, in order to promote the coating process, so the diameter of each Ag nanoparticle is determined to be about 30 nm and the diameter of each Sn nanoparticle is determined to be about 100 nm. Then, according to the literature, when the total length of the hydrophobic chain of the composite dispersant is fixed, the larger the difference in the length of the hydrophobic chain is, the stronger the dissolution capacity of the composite system will be [6]. Therefore, anionic sodium dodecylsulfate (SDS) was selected as the dispersant of nano-Ag, and cationic cetrimonium bromide (CTAB) was selected as the dispersant of nano-Sn. The mass ratio of SDS and nano-silver is 1 : 9, and the mass ratio of CTAB and nano-tin is 1 : 9. Ethanol was selected as the organic solvent due to its ability to dissolve SDS and CTAB and its high volatility.

When using anionic dispersant, the pH value should be adjusted in neutral or weak alkaline state, so that the dispersant has a higher degree of dissociation and maintain a certain thickness of the adsorption layer. Similarly, when using cationic dispersant, the pH value should be adjusted in neutral or weak acid state. The pH value of the solution is determined to be 7, so that the surface of the nano-tin is positively charged and the surface of the nano-silver is negatively charged.

**Figure 1.**
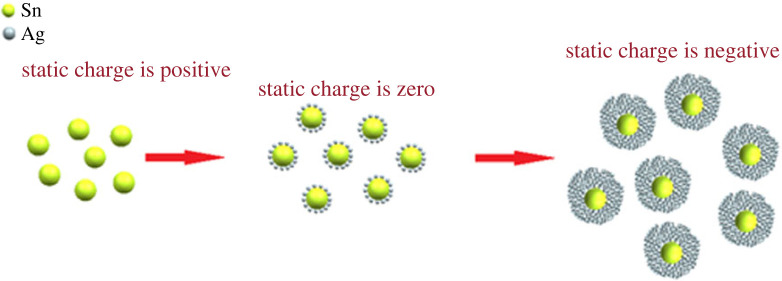
Heterogeneous flocculation process of silver-coated tin core–shell nanomaterials.

The nano-scale Ag slurry with SDS adsorbed is slowly added into the nano-scale Sn slurry with CTAB adsorbed. The two are attracted to each other by electrostatic force, and the surface charge of nano-tin changes from positive charge to zero with the increase of silver adsorption amount. As a result, the electrostatic repulsion between the rubber particles is reduced, and the thickness of the double electric layer on the surface of nano-tin is thinned. Finally, the bridge effect occurs, and heterogeneous flocculation phenomenon is generated between nano-silver and nano-tin, thus forming the nano-Sn@Ag core–shell slurry. The surface charge of the core–shell material is zero, because there is no electrostatic force and the particles are randomly distributed in the solution. Further addition of nano-Ag slurry increased the adsorption capacity of negatively charged nano-Ag, and the charge of the particles changed from zero to a higher negative value, so that the colloidal particles were re-dispersed again due to the existence of electrostatic repulsion, as shown in figure 1.

## 3. Characterization of nano-silver-coated tin slurry

**Figure 2.**
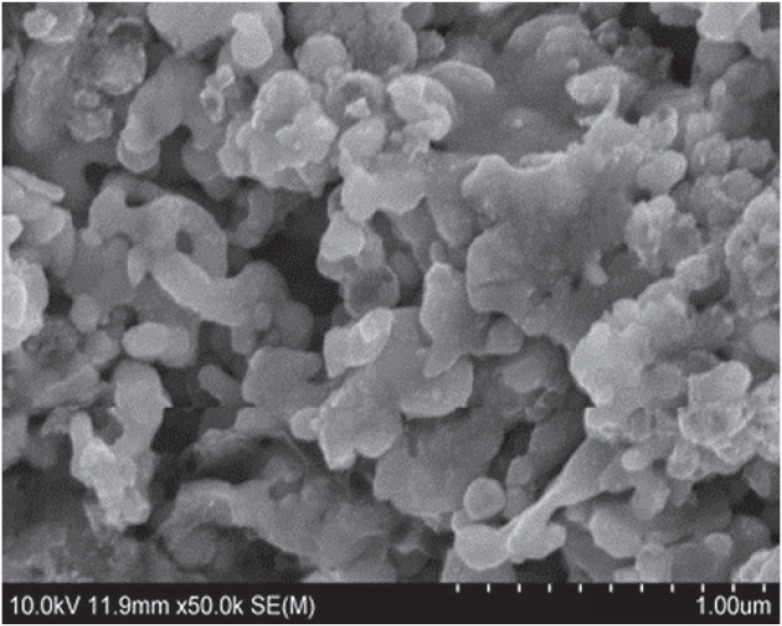
SEM image of nano-Sn@Ag paste.

**Figure 3.**
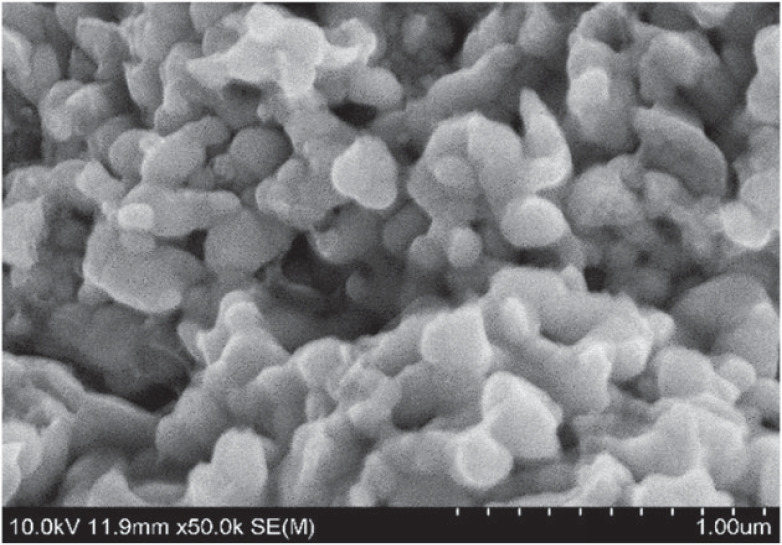
SEM image of pure nano-silver paste.

Nano-silver-coated tin slurry was prepared by heterogeneous flocculation method when the pH value of solution was 7. The SEM morphology of the prepared nano-silver-coated tin slurry is shown in figure 2. It can be seen from the figure that compared with pure nano-silver slurry (figure 3), the surface of the nanoparticles of Sn@Ag slurry prepared by nano-silver and nano-tin is obviously coated with a more uniform metal layer and has good dispersion. At this time, the content of nano-tin is 5%, which is relatively moderate. In comparison, the isoelectric point of silver nanoparticles is higher, and the anionic surfactant SDS will adsorb on silver nanoparticles. The isoelectric point of nano-tin is low, and the cationic dispersant CTAB can be adsorbed on the nano-tin particles, so that the two particles attract each other and react. The experiment shows that when the content of nanometre tin is greater than 5%, the material morphology changes, resulting in poor coating effect.

**Figure 4.**
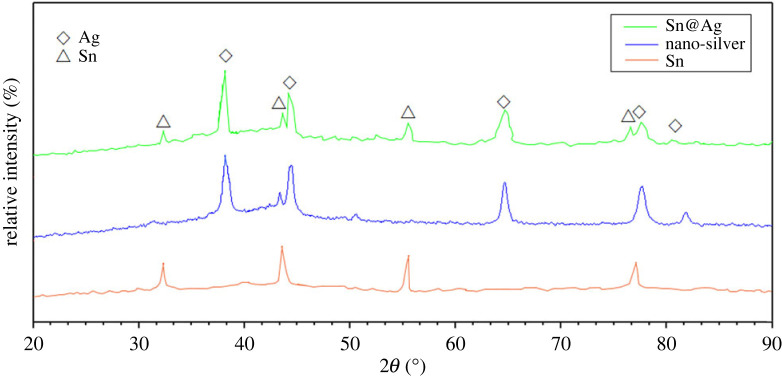
XRD curves of Cu@Ag, silver and tin nanoparticles.

Figure 4 shows the XRD pattern of the nano-core–shell material generated when the content of nano-tin is 5%. The three curves in the figure are, from bottom to top, respectively, the XRD patterns of nanometre core–shell materials generated when the diameter of tin nanoparticles is about 100 nm, silver nanoparticles with diameter of 30 nm and core–shell material when the content of tin nanoparticles is 5%. In the XRD pattern of nano-crystalline Sn@Ag, the peaks of tin and silver in the crystal structure can be clearly seen, and the impurity peaks are also less. However, the corresponding peaks of copper and silver in the curve are slightly offset from those of tin and silver nanoparticles. This is because during the formation of Sn@Ag nanoparticles, the corresponding lattice changes. At the same time, in the XRD curve of Sn@Ag nanoparticles, the peak value of silver metal is stronger, while the peak value of tin is significantly weakened compared with that of tin nanoparticles. This may be due to the formation of a tight and fully coated silver film on the surface of tin nanoparticles, thus weakening the detected characteristic peaks of tin.

## 4. Formulation of sintering process

In order to improve the sintering effect of nano-Sn@Ag slurry, a sandwich structure sample was made by using copper plate instead of substrate and chip. Similar to the sintered sample of pure nano-silver paste, the upper part is a round copper plate with a diameter of 2.8 mm, the lower part is a square copper substrate with an area of 12 × 12 mm^2^ and the middle part is connected with the upper and lower copper plates through the nano-Sn@Ag solder.

The thermal decomposition temperatures of SDS and CTAB are 250°C and 300°C, respectively. In order to fully decompose them in the sintering process, the highest sintering temperature is determined to be 300°C. The nano-Sn@Ag slurry sintering process, like the pure nano-silver slurry sintering process, can be divided into three stages. The first step is heating to 80°C and being kept for 10 min. At this time, exceeding the solvent decomposition temperature can make the ethanol fully decomposed. The second step is heating to 250°C for 10 min and it reached the decomposition temperature of anionic dispersant, so it can make SDS fully decomposed. The final step is heating to 300°C and being held for 50 min to ensure the complete decomposition of CTAB and the full diffusion and reaction of tin and silver atoms. The heating rate of sintering process is 10°C min^−1^. Sintering is carried out in air rather than vacuum, and the sintered sample is finally cooled slowly in air.

In order to study the influence of different tin contents on sintering performance, nano-Sn@Ag slurry with Sn content of 5%, 10% and 20% was prepared by the above method, and then the sintering experiment was conducted, and the sintering performance was compared with that of nano-silver slurry.

## 5. Analysis of sintering strength and morphology of nanometre Sn@Ag slurry with 5% tin content

**Figure 5.**
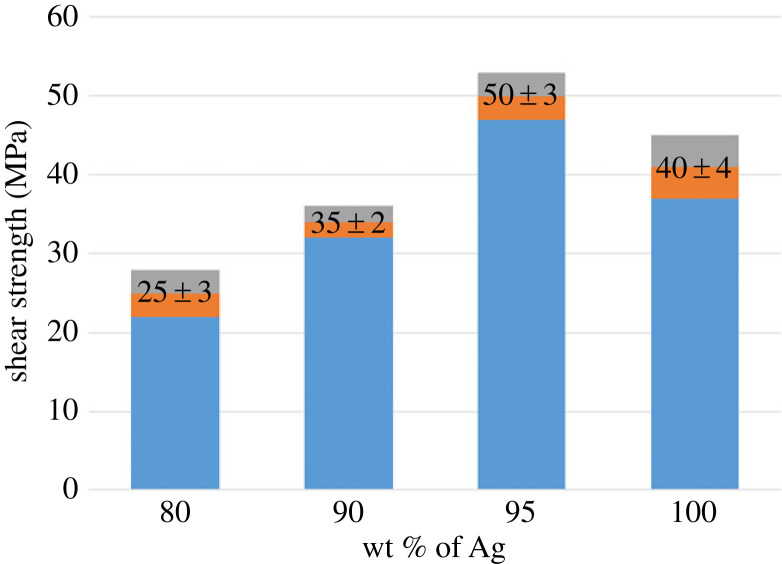
Shear strength diagram of sintered Ag + Sn joint (under 7 MPa sintering pressure).

In order to compare with the nano-Sn@Ag slurry, we first conducted a sintering experiment on the pure nano-silver slurry and used a shear tester to test the shear strength of the joint. At the sintering temperature of 300°C and the sintering pressure of 7 MPa, the average shear strength of the pure nano-silver slurry joint is 40 MPa. Then, the shear strength of the sintered joint of the nano-Sn@Ag slurry containing 5%, 10% and 20% tin content is tested. Under the same temperature and pressure, the shear strength of the Sn@Ag slurry joint with 5% tin content reaches 50 MPa, 10 MPa higher than that of the pure silver slurry joint and 15 MPa higher than that of the Sn@Ag slurry joint with 10% tin content, as shown in figure 5.

**Figure 6.**
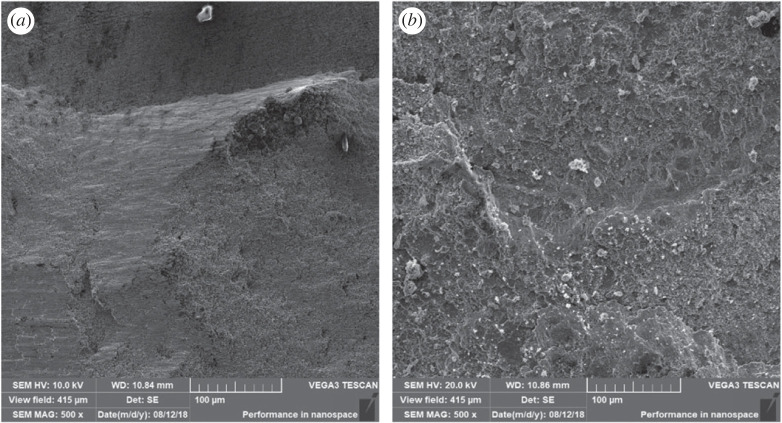
SEM images (500×) of (*a*) pure silver and (*b*) Sn@Ag nanoparticle with 5% tin content.

Figure 6 shows the 500× SEM image of Sn@Ag nanoparticle when the sintered pure silver nanoparticle and tin content is 5%. Dark areas, light areas and some holes can be seen in the image. The higher the atomic number, the lighter the colour. Tin has an atomic number of 50 and silver has an atomic number of 47, so tin is lighter than silver. Figure 6*a* is the image of sterling silver sintering layer, so the colour is darker. Figure 6*b* is the image of sintered nano-Sn@Ag slurry. In the figure, the content of light-coloured area increases significantly. The pores are formed by the breakdown of organic polymers.

**Figure 7.**
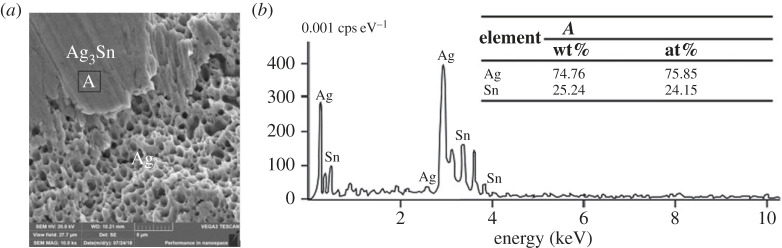
Microstructure and composition analysis of the nano-Sn@Ag slurry joint with 5% Sn content. (*a*) SEM image of Sn@Ag slurry joint with 5% tin content and (*b*) local EDS analysis.

Figure 7*a* is the SEM image of the nanometre Sn@Ag slurry joint with 5% tin content. Compared with the dark sintering morphology of pure nano-silver slurry, it can be found that the sintering morphology of nano-Sn@Ag slurry is alternated between light and dark, in which the light-coloured area is sintered denser, and the composition analysis of the denser area *A* is shown in figure 7*b*. It shows that the content of Ag atoms is 3 times that of Sn atoms, which is the same as the atomic ratio of compound Ag_3_Sn, so it can be inferred that the final product of sintered nano-Sn@Ag slurry is composed of Ag and Ag_3_Sn, and this mixture can promote the sintering performance of nano-Sn@Ag slurry. The absence of oxygen in the energy spectrum indicates that the solder forms a core–shell structure and the Ag shell coating Sn core can prevent Sn oxidation.

## 6. Research on interconnection mechanism

It is helpful to analyse the sintering mechanism of the nano-Sn@Ag slurry (silver–tin slurry) by mastering the phase change of the slurry due to the temperature increase. The phase variation and the meso-morphology of sintered joint can be estimated by the silver–tin binary phase diagram. The phase diagram shows the composition of different solubility, so it is possible to predict the sintered phase of silver–tin slurry at various temperatures [7].

**Figure 8.**
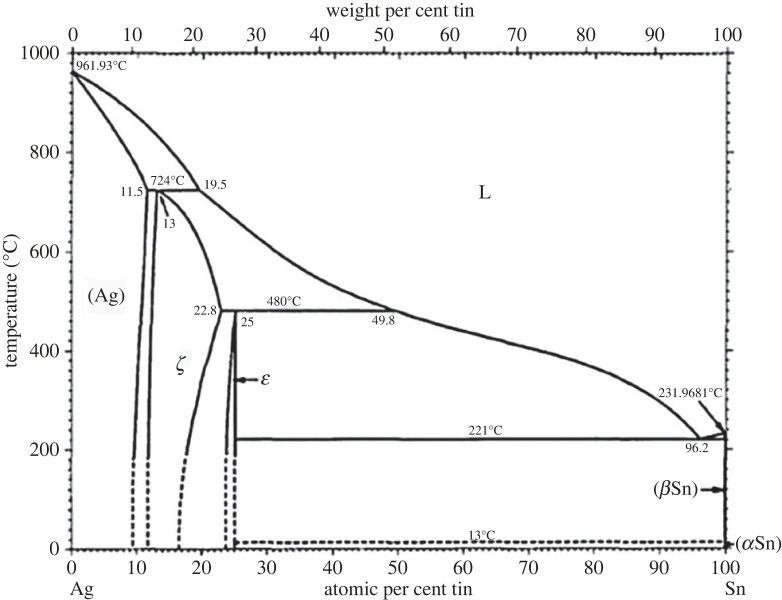
Phase diagram of silver–tin binary alloy [8].

As shown in figure 8, the melting point of Ag is 962°C and that of Sn is 232°C. The process obviously belongs to the transient liquid phase sintering; the silver proportion is high, belonging to the matrix material, the tin proportion is low, belonging to the doping material. In figure 8, the content of tin is 25%, which is in Ag–Sn solid solution [8]. With sintering atom diffusion, diffusion is the phenomenon of two different concentrations of metal atoms transferring to each other, general alloy solder, mutual diffusion belonging to the mainstream form.

The beginning of sintering is the diffusion between solids. When the metal tin melts into a liquid, the liquid tin will continue to diffuse into the matrix silver until the end of all the liquid phase transformation at the time of solidification [9]. So, the transient liquid phase method is complicated and multi-stage. Ag reacts with Sn to form Ag_3_Sn, which then forms the final equilibrium phase. The sintering method has the following characteristics: (i) it can reduce the sintering temperature of the sample; (ii) the sintering strength of the sample increases as Sn diffuses into the matrix Ag; and (iii) the joint can be used at higher temperatures.

**Figure 9.**
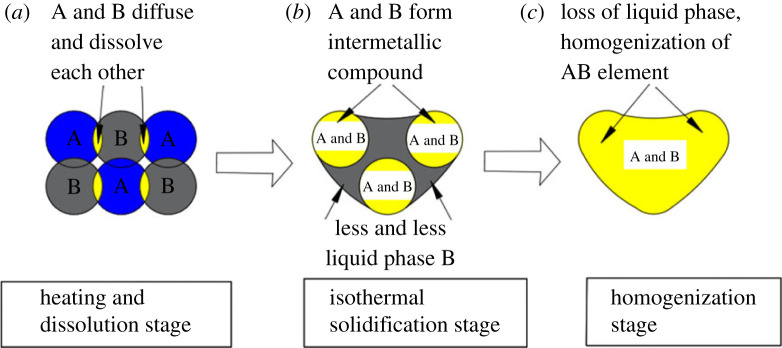
(*a*–*c*) Transient liquid phase sintering process.

The change of sintering temperature of silver–tin slurry affects the transformation of phase morphology. According to the silver–tin phase diagram, the interconnect process of silver–tin slurry is divided into three stages by sintering temperature [10]. The sintering mechanism of silver–tin slurry can be explained according to the transient liquid phase sintering process in figure 9.

The sintering temperature of the first stage of the transient liquid phase method is between 25 and 300°C. When the temperature rises, the mutual diffusion of Ag particles and Sn particles begins, and the new phase will be gradually generated in this process. According to the silver–tin phase diagram, eutectic reaction will occur when the sintering temperature reaches 221°C. As the transient liquid phase sintering continues, the amount of liquid tin produced gradually increases [11,12]. When the liquid tin contacts the solid silver, the liquid will rise or penetrate along the gap, causing the Ag matrix to become tight. Where there are more solid Ag particles, silver–silver diffusion occurs between Ag particles. Where Ag–Sn particles are present, the degree of mutual diffusion of Ag–Sn also increases, resulting in a large amount of Ag dissolved in liquid Sn. Figure 9*a* shows the process.

The sintering temperature of the second stage of the transient liquid phase method is 300°C, which is the stage of isothermal solidification. When Ag particles continuously enter the liquid Sn and accumulate to a certain amount, they will react to form an intermetallic compound, the melting point of which is proportional to the solubility of Ag [13,14]. The liquid-phase reaction results in a giro phase (Ag_3_Sn) when the solubility of Ag in Sn is 75%. The melting point of Ag_3_Sn is 480°C, much higher than that of Sn. The generated liquid phase will gradually diffuse into the silver matrix. According to the trend of the silver–tin phase diagram, the liquid constantly changes into a solid and finally gradually disappears. At the same time, solid Ag also completes the sintering step, as shown in figure 9*b*.

The third stage of transient liquid phase method is insulation at 300°C, which is the homogenization stage. Over time, Ag–Sn atoms are further interdiffused, resulting in uniform concentrations everywhere. At the end of sintering, the equilibrium phase formed is Ag–Sn replacement solid solution and Ag_3_Sn, which play the roles of solid solution strengthening and dispersion strengthening, respectively [15,16], as shown in figure 9*c*.

## 7. Summary

Based on the theory of transient liquid phase sintering, a new process for preparing silver–tin nano-core–shell materials was proposed in this paper. By means of adjusting the pH value of the solution and selecting different types of dispersing agents, the nano-silver-coated tin slurry was successfully prepared by adopting heterogeneous flocculation method. By this method, the sintering temperature of nano-silver slurry can be reduced, the shear strength can be increased and the oxidation resistance and dispersion of tin in the silver matrix can be improved. When the sintering temperature is 300°C, the sintering strength of Sn@Ag slurry increases with the increase of Sn content. When the Sn content reaches 5%, the interconnection strength of the joint reaches the highest 50 MPa, which is more than 10 MPa higher than that of the pure nano-Ag slurry sintered joint. The analysis of the nano-Sn@Ag slurry sintered joint shows that the improvement of interconnect strength is due to the formation of equilibrium phase Ag–Sn replacement solid solution and intermetallic compound Ag_3_Sn, which have the effects of replacement solution strengthening and dispersion strengthening, respectively.

## Data Availability

The data of this study are available from the corresponding author upon reasonable request.
